# Identifying Gastric Cancer Related Genes Using the Shortest Path Algorithm and Protein-Protein Interaction Network

**DOI:** 10.1155/2014/371397

**Published:** 2014-03-05

**Authors:** Yang Jiang, Yang Shu, Ying Shi, Li-Peng Li, Fei Yuan, Hui Ren

**Affiliations:** ^1^Colorectal Surgery Department, China-Japan Union Hospital of Jilin University, Changchun 130033, China; ^2^State Key Laboratory of Medical Genomics, Institute of Health Sciences, Chinese Academy of Sciences, Shanghai Jiao Tong University School of Medicine and Shanghai Institutes for Biological Sciences, Shanghai 200025, China; ^3^Breast and Thyroid Surgery Department, The Second Hospital of Jilin University, Changchun 130041, China; ^4^Colorectal Surgery Department, The Second Hospital of Jilin University, Changchun 130041, China

## Abstract

Gastric cancer, as one of the leading causes of cancer related deaths worldwide, causes about 800,000 deaths per year. Up to now, the mechanism underlying this disease is still not totally uncovered. Identification of related genes of this disease is an important step which can help to understand the mechanism underlying this disease, thereby designing effective treatments. In this study, some novel gastric cancer related genes were discovered based on the knowledge of known gastric cancer related ones. These genes were searched by applying the shortest path algorithm in protein-protein interaction network. The analysis results suggest that some of them are indeed involved in the biological process of gastric cancer, which indicates that they are the actual gastric cancer related genes with high probability. It is hopeful that the findings in this study may help promote the study of this disease and the methods can provide new insights to study various diseases.

## 1. Introduction

Gastric carcinogenesis is a multistep process involving genetic and epigenetic alteration of protein-coding protooncogenes and tumor-suppressor genes. Gastric cancer (GC) is the fourth most commonly diagnosed cancer and is estimated to be the second most common cause of cancer related death and causes about 800,000 deaths worldwide per year [1, 2]. Because of the improvement of the dietary structure, the mortality rate shows a declining trend worldwide [3]. However, the incidences of gastric cancer are still remarkable in areas where infection by *Helicobacter pylori* is prevalent [4]. Besides *H. pylori*, smoking and alcohol consumption also increase the risk of developing gastric cancer significantly [5, 6]. Compared with women, men have a higher incidence, while estrogen may protect women against the gastric cancer [7].

In the previous cases, over 90% gastric cancers are adenocarcinomas, which could be divided into two major types in terms of the histopathology [8]. Intestinal type gastric cancer is often related to environmental factors such as *H. pylori*, while diffuse type gastric cancer is more often associated with genetic abnormalities. Caldas et al. reviewed that the diffuse type gastric cancer tended to occur in female and young individuals [9]. Besides adenocarcinomas, other types of gastric cancers like lymphomas occurred in a very low incidence [10]. Since the gastric cancer leads to high mortality, the early diagnose especially the molecular diagnose is particularly important for the therapy.

So far, numerous genes have been found involved in gastric tumorigenesis. Among the reported gastric cancer related genes, most of them could have also been found in other types of carcinomas. p53, famous for its tumor-suppressing role, has a mutated rate ranging from 0 to 21% in diffuse type GC and 36–43% in intestinal type GC [11]; E-cadherin, which plays a pivotal role in EMT (Epithelial Mesenchymal Transition), is predisposed to mutagenesis in sporadic diffuse type GC (33–50%) [12]; another star gene harboring high correlation with gastric cancer is RNUX3, which manifests to be a tumor-suppressor gene of GC [13]. Although dozens of genes have been found related to gastric cancer, they are insufficient to elucidate the tumorigenesis of GC unless more relevant genes being uncovered.

It is time-consuming to discover novel gastric cancer related genes by experiment alone, because the search space is very large. Computational approach is an alternative way which can help investigators screen out some related genes. On the other hand, lots of computational approaches have been developed to settle various biological problems, such as drug design [14–19] and analysis of complicated biological network [20–24]. In this study, a computational method was built to discover novel gastric cancer related genes based on some known related ones retrieved from Gastric Cancer Database, UniProtKB, and TSGene Database. After applying the shortest path algorithm in protein-protein interaction network to search the shortest path connecting any pair of known gastric cancer related genes, the candidate genes were found. Further analysis suggests that some of them are related to the formation and development of gastric cancer. We hope that this contribution may give help to uncover the mechanism of this disease, thereby designing effective treatments.

## 2. Materials and Methods

### 2.1. Materials

Gastric cancers related genes are collected from the following three datasets: (1) 102 genes are picked up from Gastric Cancer Database (http://www.gastric-cancer.site40.net/); (2) 128 reviewed gastric cancer related genes were found in the UniProtKB (Protein Knowledgebase, http://www.uniprot.org/uniprot/) by setting the keyword as human gastric cancer oncogene/suppressor gene, where 86 are oncogenes and 42 are suppressor genes; (3) 9 genes were obtained from TSGene Database (Tumor Suppressor Gene Database, http://bioinfo.mc.vanderbilt.edu/TSGene/) by searching the human gastric cancer in the Literature Search box. After combining these genes, we obtained 150 gastric cancer related genes, which were available in Supplementary Material I (available online at http://dx.doi.org/10.1155/2014/371397).

### 2.2. Protein-Protein Interaction (PPI) Network

It is known that interactions of proteins are important for the majority of biological functions. Many studies have shown that proteins in one interaction always share similar functions [25–29]. Since gastric cancers related genes may have some common features, it is feasible to discover novel gastric cancers related genes based on known related ones and PPI network. In this study, the PPI network was constructed based on the protein interaction information retrieved from STRING (Search Tool for the Retrieval of Interacting Genes/Proteins, http://string.embl.de/) (version 9.0) [30], a well-known database integrating known and predicted protein interactions. In the obtained file, each interaction consists of two protein IDs and a score measuring the likelihood of the interaction's occurrence. For later formulation, the score of the interaction between proteins *p*_1_ and *p*_2_ was denoted by *I*(*p*_1_, *p*_2_). To construct the weighted network, proteins in the STRING were taken as nodes and two nodes were adjacent if and only if the score of the interaction between the corresponding proteins was greater than zero. In addition, the score of the interaction was used to label the weight of the corresponding edge as follows:

(1) $$\begin{array}{c} w\left(v_{1},v_{2}\right)=1000-I\left(p_{1},p_{2}\right), \end{array}$$

where *p*_*i*_  (*i* = 1,2) was the corresponding protein of node *v*_*i*_.

### 2.3. Shortest Path Genes

As described in Section 2.1, 150 gastric cancer related genes were collected, which must have some common features related to gastric cancer. On the other hand, according to Section 2.2, two proteins in one interaction, that is, they are adjacent in the constructed PPI network, always share common features. It can be further deduced that proteins in the shortest path connecting two known gastric cancer related genes may share some common features that the two known gastric cancer related genes have. Therefore, we searched the shortest path between any pair of known gastric cancer related genes by Dijkstra's algorithm, the most famous shortest path algorithm proposed by Dijkstra in 1956 [31].

After collecting the shortest paths connecting any pair of known gastric cancer related genes, we found that some nodes/genes occurred in many paths, while the majority of nodes/genes in PPI network were not in any path. To distinguish these nodes/genes, the betweenness of each node/gene was calculated, which is defined as the number of the shortest paths containing the node/gene as an inner node. Since the concept of betweenness accounts for direct and indirect influences of proteins at distant network [32], it has been employed in the study of various natural and man-made networks [33–38].

It is easy to see that genes with high betweenness may share more features related to gastric cancer than those with low betweenness, while the likelihood of gene with betweenness equal to 0 to be the novel gastric cancer related gene is zero. Accordingly, we picked out genes with betweenness greater than 0 and termed them as the shortest path genes. Since the main purpose of this study is to discover novel gastric cancer related genes, the known gastric cancer related genes were not included in the set of shortest path genes.

### 2.4. Further Filtering Based on Permutation Test

As described in Section 2.3, some of the shortest path genes can be obtained based on their betweenness. However, the betweenness of some nodes may be strongly influenced by the essential structure of the network. For example, the cut-vertex of the network may always receive high betweenness easier than other vertices. To control this false discovery, a permutation test was conducted to further filter these shortest path genes as follows.

(i) Randomly select 1,000 gene sets *G*_1_, *G*_2_,…, *G*_1000_ in PPI network with the same size of known gastric cancer related gene set.
(ii) Calculate the betweenness of each shortest path gene on each gene set *G*_*i*_  (1 ≤ *i* ≤ 1000).
(iii) The permutation FDR of the shortest path gene *p* was computed by
  (2) $$\begin{array}{c} \text{FDR}\left(p\right)=\frac{\sum_{i=1}^{1000}\delta_{i}}{1000}, \end{array}$$

where *δ*_*i*_ was defined to be 1 if the betweenness of *p* on *G*_*i*_ was greater than that of *p* on the known gastric cancer related gene set.

It is obvious that smaller permutation FDR of one shortest path gene indicates that it is the actual gastric cancer related gene with high possibility.

### 2.5. Gene Set Enrichment Analysis

DAVID [39] is a functional annotation tool, which has been widely used to analyze gene lists derived from different biological problems [40–45]. Here, it was also employed for KEGG pathway and GO enrichment analysis of the obtained gene set. The enrichment *P* value was corrected to control family-wise false discovery rate under certain rate (e.g., ≤0.05) with Benjamin multiple testing correction method [46]. During the enrichment analysis, all genes in the human genome were considered. 13 items in the output of DAVID and their meanings are listed in Table 1. For detailed description, please see Huang et al.'s study [39].

## 3. Results and Discussion 

### 3.1. Candidate Genes

Of the 150 known gastric cancer related genes, the shortest path connecting any pair of them was searched in PPI network constructed in Section 2.2. After counting the betweenness of each gene in PPI network, 466 shortest path genes with betweenness greater than zero were retrieved. These 466 genes and their betweenness can be found in Supplementary Material II. To exclude the false discovery, the permutation test was conducted. The permutation FDRs of 466 shortest path genes were calculated by (2) and also listed in Supplementary Material II. It can be observed that 144 genes were with permutation FDRs no more than 0.1. These genes were considered to have a strong relationship with gastric cancer.

### 3.2. Results of Gene Set Enrichment Analysis

DAVID, as a functional annotation tool, was employed to analyze the 144 shortest path genes. The analysis results included two categories: GO and KEGG. These results were available in Supplementary Materials III and IV, respectively. The detailed discussion based on these results was as follows.

**Figure 1:**
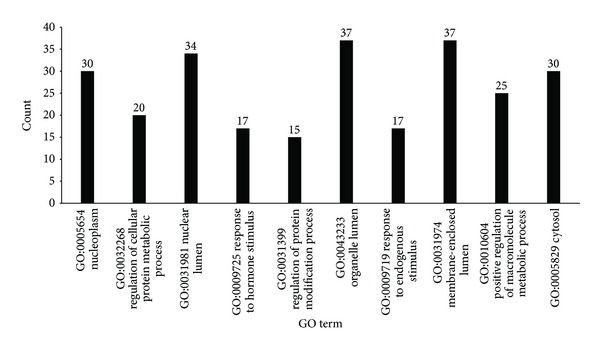
The top 10 GO terms enriched by 144 genes. The *x*-axis lists GO's ID and name, while the *y*-axis represents the number of genes that shared the GO term among the 144 genes.

From Supplementary Material III, 294 GO terms were enriched by the 144 genes. We investigated the first 10 GO terms in the list, which were shown in Figure 1. The “Count” items in the output of DAVID for these 10 GO terms were also shown in Figure 1. Among these 10 GO terms, 5 out of the 10 GO terms are cellular component (CC) GO terms including (1) GO:0005654: nucleoplasm (“count” = 30); (2) GO:0031981: nuclear lumen (“count” = 34); (3) GO:0043233: organelle lumen (“count” = 37); (4) GO:0031974: membrane-enclosed lumen (“count” = 37); (5) GO:0005829: cytosol (“count” = 30). As we know, tumorigenesis is a very complicated biological process which means the transform processes could take place everywhere in the cells [47]. In our analysis results, the related proteins distribute both in nuclear and cytosol which is in accordance with the characters of the gastric cancer. The remaining 5 GO terms are biological process (BP) GO terms: (1) GO:0032268: regulation of cellular protein metabolic process (“count” = 20); (2) GO:0009725: response to hormone stimulus (“count” = 17); (3) GO:0031399: regulation of protein modification process (“count” = 15); (4) GO:0009719: response to endogenous stimulus (“count” = 17); (5) GO:0010604: positive regulation of macromolecule metabolic process (“count” = 25). Liu et al. reported that the cancer cells usually harbor abnormal metabolic status [48]. In our results 80% (4/5) BP are relative to the metabolic stress response by means of direct regulation of the metabolic process or indirect regulation by altering the stimulus-related pathways. Besides, protein modification, which is also enriched in our results, plays an important role in the carcinogenesis by altering the pivotal proteins [49]. Although these genes may not be the indispensable factors in gastric cancer, the common points among them would give us the hints about the tumorigenesis of the gastric cancer.

**Figure 2:**
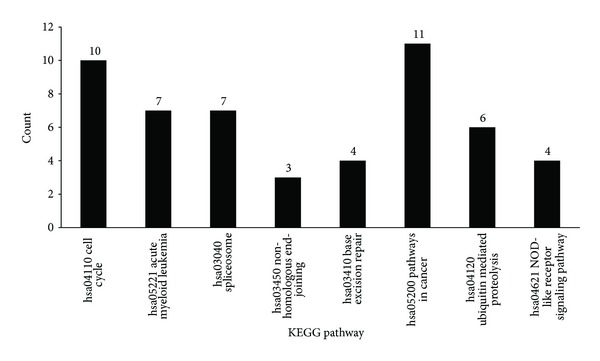
The 8 KEGG pathways enriched by 144 genes. The *x*-axis lists pathway's ID and name, while the *y*-axis represents the number of genes that shared the pathway among the 144 genes.

From Supplementary Material IV, 8 KEGG pathways were enriched by 144 genes, which were shown in Figure 2. It can be observed that 6 out of 8 KEGG pathways were with *P* value less than 0.05, which were investigated as follows. The first pathway was hsa04110: cell cycle pathway (“count” = 10). 10 genes including PCNA, MYC, and CCND1 are enriched in this pathway. One of the significant characters of gastric cancer is the abnormal activated cell cycle [50]. Among these genes, PCNA is responsible for the DNA synthesis and CCND1 could alter cell cycle by regulating the CDK kinases [51, 52]. Other 2 pathways found in our study are related to the DNA repair which are also very critical for the carcinogenesis. Hsa03450: nonhomologous end joining (NHEJ) (“count” = 3) is a pathway that repairs double-strand breaks in DNA and base excision repair (BER) is a cellular mechanism that repairs damaged DNA throughout the cell cycle [53, 54]. Another intriguing pathway is hsa03040: spliceosome pathway (“count” = 7) which was always abnormal in cancer cells [55]. We speculate that the spliceosome could modify the expression of the oncogenes or tumor-suppress genes which eventually lead to the tumorigenesis. Finally, we also find the hsa05221: acute myeloid leukemia (AML) pathway (“count” = 7) and cancer related pathways in our list. The results imply that the gastric cancer has the common mechanism as well as other cancers especially the AML. Look has reviewed that RUNX1 is the key factor in the hematopoietic development and highly correlated with AML [56]. However, its homologous protein RUNX3 that shares 70% similarity has been reported playing pivotal role in gastric cancer [57]. The finding unravels that cancer normally has the common molecular mechanism as well as the specific pathway with type-dependent pattern. Although several reported pathways are included in our study, the novel pathways with gastric cancer would expand our views of mechanisms about the tumorigenesis of gastric cancer. On the other hand, we have observed that some genes in these pathways could play a very important role in the carcinogenesis of gastric cancer.

### 3.3. Analysis of the Relationship of Some Candidate Genes and Gastric Cancer

As described in Section 3.1, 144 genes were discovered by our method. Some of them may have strong relationship with gastric cancer and were discussed as follows.  Table 2 listed these genes and their betweenness and permutation FDRs.

Proliferating Cell Nuclear Antigen (PCNA) (see row 2 of Table 2), also known as cyclin, is an auxiliary protein of DNA polymerase-*δ* that plays important roles both in DNA synthesis and DNA repair [51, 58]. PCNA could act as a homotrimer and helps increase the processivity of leading strand synthesis during DNA replication [59, 60]. In response to DNA damage, this protein is ubiquitinated and is involved in the RAD6-dependent DNA repair pathway [61, 62]. As we know, DNA repair is the main way to remove the carcinogenic lesions caused by UV or other common mutagens [63]. Pascucci et al. have reviewed that the NER (nucleotide excision repair) was highly correlated with skin cancer and intestinal cancer [64]. Intriguingly, numerous works have considered PCNA labeling rate as the prognostic indicator of gastric cancer because its expression was consistent with malignant potential of gastric cancer [65–67]. Ji et al. have found the abnormal increase of PCNAexpression in 58 gastric carcinoma tissues [68]. Similar conclusion was also achieved by Takamura et al. who have performed immunohistochemical study on 164 patients with gastric carcinomas [69]. Although the strong correlation is observed between PCNAand gastric cancer, the detailed mechanism of how PCNA promotes the gastric cancer needs further elucidation.

Besides PCNA, another protein in highly conserved cyclin family was also found in our study. CCND1 (see row 3 of Table 2), with official full name of cyclin D1, was firstly described by Motokura et al. in 1991 [70]. In the following decades, the importance of CCND1 in cell cycle and tumorigenesis was underlined by different labs. Because of the amplification of the 11q13 region where CCND1 locates, CCND1 is frequently overexpressed in human cancers accompanied with abnormalities that are driven by multiple mechanisms including genomic alternations, posttranscriptional regulation and posttranslational protein stabilization [71–73]. On one hand, cyclin D1 could increase CDK activity and consequently result in continuous proliferation which is necessary for tumorigenesis [74, 75]. On the other hand, cyclin D1 may induce the tumorigenesis in certain types of cancers by means of its nuclear receptor-agonistic activity in the CDK-independent way [52, 76].

MYC (see row 4 of Table 2) is a regulator gene that codes for a transcription factor, and it is frequently mutated in many cancers. In Myc-related cancers, Myc is constitutively expressed and leads to the abnormal expression of many genes which may be involved in cell proliferation, differentiation and apoptosis, and these uncontrolled biological processes finally underlie the cancer. Myc is believed to regulate expression of 15% of all genes [77]. Similar with CCND1, Myc expression could be regulated transcriptionally, posttranscriptionally, or posttranslationally [78]. Chung and Levens have reviewed that the deregulated expression of Myc is sufficient to lead to cellular transformation *in vitro* and tumorigenesis *in vivo* [79]. Besides the transforming role, Myc could also promote chromosomal instability by means of its function as a transcriptional regulator [80]. In the previous reports, Myc overexpression has been described in over 40% of gastric cancer [81]. Among nearly half the gastric cancer, copy number gains are frequently detected along chromosome 8 where Myc locates [82, 83]. As the key factor of tumorigenesis, Myc could provide potential target for therapy for gastric cancer [84].

FOS (see row 5 of Table 2), well known as c-fos, encodes a 62 kDa protein, which forms heterodimer with c-jun and subsequently results in the formation of AP-1 complex. FOS has been found to be overexpressed in a variety of cancers. Bakin and Curran have found that c-fos could change DNA methylation pattern by regulating DNMT1 and thereby cause the downregulation of tumor suppressor genes [85]. In addition, c-fos could lead to the loss of cell polarity and EMT which is critical for the metastatic and invasive growth of cancer cells [86]. Hu et al. also found that c-fos is required for the expression of matrix metalloproteinases that are indispensable for invasive growth of cancer cells [87]. However, some recent studies have unraveled the tumor suppressor activity of c-fos, including prohibition of the cell cycle progression, promotion of cell death, or repressing the anchorage-independent growth [88]. In coincidence with the negative role of c-fos in tumorigenesis, Jin et al. analyzed 625 consecutive gastric cancers; 388 cases (62.1%) showed loss of nuclear c-fos expression [89]. Consistent results were concluded by Zhou et al. in 58 patients with gastric cancer [90]. However, Mazurenko et al. reported that high level of c-fos expression was observed in stomach carcinomas [91]. The discordance may be caused by the different stages of progression in different studies. In conclusion, c-fos is a double-edged sword, which could promote or suppress tumorigenesis of gastric cancer.

RUNX1 (see row 6 of Table 2), better known as AML1, plays a critical role in hematopoietic development [92]. RUNX1 belongs to the RUNX family whose 3 members (RUNX1, RUNX2, and RUNX3) share 70% resemblance. Unlike its familial protein RUNX3 that is a strong candidate as a gastric cancer tumor suppressor. RUNX1 is always considered as a tumor suppressor for acute lymphoblastic leukemia (AML) [56]. Usui et al. have examined mRNA expression of all three RUNX genes in the gastric mucosa, and they found that RUNX1 was coexpressed with RUNX3 in pit cells [93]. Sakakura et al. observed remarkable downregulation of RUNX1 and RUNX3 in 9 gastric cancer cell lines and 56 primary gastric cancer specimens [94]. Although RUNX1 is famous for its involving in AML, more lines of evidence shed light to its anticarcinogenesis activity in other carcinoma including gastric cancer.

Other genes found in our study have also been reported relating with gastric cancer. Specific SNPs (Single Nucleotide Polymorphism) in XRCC1 (X-ray repair cross-complementing 1) (see row 7 of Table 2) are highly associated with gastric cancer [95]. DNMT1 (DNA methyltransferase 1) (see row 8 of Table 2), which is overexpressed in gastric cancer, is associated with increased risks of gastric atrophy with its abnormal polymorphisms [96]. The expression of CXCL1 (chemokine (C-X-C motif) ligand 1) (see row 9 of Table 2) is higher in gastric cancer tissues and endows the cancer cells with more powerful migration and invasion ability [97]. Beyond these genes, more genes associated with gastric tumorigenesis require more evidences for validation or further exploration.

## 4. Conclusion

Identification of disease genes is one of the most important problems in biomedicine and genomics. For gastric cancer, as one of the leading causes of cancer related deaths worldwide, it is eager to discover its related genes, which can help to uncover its mechanism and design effective treatments. This contribution presented a computational method to identify novel gastric cancer related genes based on known related ones by shortest path algorithm and PPI network. The analysis implies that some genes discovered in this study have direct or indirect relationship with gastric cancer. It is hopeful that this contribution would give a new insight to study this disease and other diseases.

## Supplementary Material

The Supplementary Material consists of four files. In detail, Supplementary Material 1 lists 150 gastric cancer related genes; Supplementary Material 2 lists the shortest path genes and their permutation FDRs; Supplementary Material 3 lists GO enrichment results of 144 genes; Supplementary Material 4 lists KEGG enrichment results of 144 genes.

## Figures and Tables

**Table 1:** Items in the output of DAVID and their meanings.

Item	Meaning
Category	DAVID category, that is, KEGG or GO
Term	Gene set name
Count	The number of genes associated with this gene set
Percentage	Calculated by “gene associated with this gene set”/“total number of query genes”
*P* value	Modified Fisher Exact *P* value
Genes	The list of genes from your query set that are annotated to this gene set
List total	The number of genes in your query list mapped to any gene set in this ontology
Pop hits	The number of genes annotated to this gene set on the background list
Pop total	The number of genes on the background list mapped to any gene set in this ontology
Fold enrichment	The ratio of the proportions on query genes and the background information which are associated with the gene set
Bonferroni	Bonferroni adjusted *P* value
Benjamini	Benjamini adjusted *P* value
FDR	FDR adjusted *P* value

**Table 2:** Important candidate shortest path genes and their betweenness and permutation FDRs.

Ensemble ID of shortest path genes	Gene name	Betweenness	Permutation FDR
ENSP00000368438	PCNA	454	0.083
ENSP00000227507	CCND1	594	0.02
ENSP00000367207	MYC	779	0.01
ENSP00000306245	FOS	318	0.035
ENSP00000300305	RUNX1	224	0.002
ENSP00000262887	XRCC1	152	0.094
ENSP00000352516	DNMT1	169	0.093
ENSP00000379110	CXCL1	107	0.033
