# Supplementary material for: Identifying Gastric Cancer Related Genes Using the Shortest Path Algorithm and Protein-Protein Interaction Network
Source: Biomed Res Int. 2014 Mar 5;2014:371397. doi: 10.1155/2014/371397 (PMC3963223; doi:10.1155/2014/371397)
Supplement: Supplementary file 1 — The Supplementary Material consists of four files. In detail, Supplementary Material 1 lists 150 gastric cancer related genes; Supplementary Material 2 lists the shortest path genes and their permutation FDRs; Supplementary Material 3 lists GO enrichment results of 144 genes; Supplementary Material 4 lists KEGG enrichment results of 144 genes. [file 371397.f1.pdf]

Supplementary Material I. 150 Gastric cancers related genes collected from Gastric Cancer Database, UniProtKB and TSGene Database

ADRM1	FAM120A	PIM3
AKAP12	FAM72D	PIWIL1
AKAP9	FGFR2	PLK1
ANPEP	FGFR3	PLXNB1
APC	FGFR4	PPHLN1
ARID1A	FLNC	PQBP1
ATM	GKN2	PRDM5
AXL	GRB7	PRKAA2
BACE2	GSDMA	PRSS1
BIRC5	HDAC1	PSCA
BMPR1A	HMGB1	PTPN23
BMPR1B	HNRPDL	RARRES3
BRD3	HPGD	ROS1
BRDT	INSR	RPS6KA3
BRSK1	IRF1	RRP7A
CA9	JAK3	RTKN
CAPRIN2	JMJD6	RUNX3
CASC3	KCMF1	SAPCD2
CASP10	KIAA1524	SDHB
CASP8	KISS1	SDHD
CASP9	KLF6	1-Sep
CCKBR	KRAS	SERF2
CDC23	LDHA	SKI
CDCA8	LGALS3	SLC5A8
CDH1	LOX	SMAD2
CDK12	LXN	SMAD3
CENPW	LZTS1	SPEG
CHFR	MAP3K9	STEAP4
CIAO1	MAPK14	STK11
CIB1	MDM4	TACC1
CKAP2	MET	TAOK3
CLU	MICB	TCF7L2
COPA	MINK1	TCHP
CTAG2	MLH1	TERT
DLC1	MSH2	TFF1
DLEC1	MST4	TGFBR2
DMBT1	MTAP	TLK2
DUS2L	MUTYH	TMEFF2
EIF3D	MYEOV	TNK2
EIF3K	NAA15	TP53
EIF4E	NDFIP1	TRAF2

ELF3	NEK6	TRRAP
EMCN	NENF	TTN
ENO1	NOL8	UBAP1
EPHA2	NTRK3	UBD
EPHA8	NUCB2	VIM
EPHB1	OGG1	WISP1
EPHB2	PDGFRB	WNT10B
EPHB4	PDX1	WWOX
ERBB2	PIK3CA	ZFHX3
