# Supplementary material for: Identifying Gastric Cancer Related Genes Using the Shortest Path Algorithm and Protein-Protein Interaction Network
Source: Biomed Res Int. 2014 Mar 5;2014:371397. doi: 10.1155/2014/371397 (PMC3963223; doi:10.1155/2014/371397)
Supplement: Supplementary file 2 [file 371397.f2.pdf]

Supplementary Material II. 466 shortest path genes with betweenness greater than 0 and their permutation FDRs

Ensemble ID of shortest path genes	Gene name	Betweenness	Permutation FDR
ENSP00000245323	EFNB2	643	0
ENSP00000415151	CBFB	158	0
ENSP00000269601	TXNL4A	148	0
ENSP00000301411	NTF4	27	0
ENSP00000313420	PRKDC	286	0.001
ENSP00000300305	RUNX1	224	0.002
ENSP00000338548	FGF1	304	0.003
ENSP00000265814	RUNX1T1	214	0.003
ENSP00000376849	CASP5	152	0.003
ENSP00000307298	FEM1B	40	0.003
ENSP00000313950	AURKB	339	0.004
ENSP00000216911	AURKA	307	0.004
ENSP00000219172	CENPT	152	0.004
ENSP00000412553	ABCF1	152	0.004
ENSP00000388107	UBA52	561	0.005
ENSP00000222005	CDC37	304	0.005
ENSP00000352257	XRCC6	153	0.005
ENSP00000222482	CPA4	152	0.007
ENSP00000372975	HLA-C	152	0.007

ENSP00000234371	KISS1R	151	0.007
ENSP00000339723	CIR1	151	0.007
ENSP00000397297	NTF3	125	0.007
ENSP00000357392	EFNA1	174	0.008
ENSP00000239440	ARAP3	152	0.008
ENSP00000307004	APLF	152	0.009
ENSP00000355890	EPRS	152	0.009
ENSP00000358617	PHTF1	2	0.009
ENSP00000367207	MYC	779	0.01
ENSP00000229268	USP5	152	0.01
ENSP00000274026	CCNA2	411	0.011
ENSP00000253108	EIF3G	151	0.012
ENSP00000295926	CCNL1	152	0.013
ENSP00000338207	LMO1	150	0.013
ENSP00000240618	KLRK1	303	0.014
ENSP00000340691	EIF4EBP1	235	0.014
ENSP00000372815	C4A	152	0.015
ENSP00000373487	KRT18	152	0.015
ENSP00000350941	SRC	1283	0.016
ENSP00000206542	OSGEP	152	0.016
ENSP00000380460	PLAA	112	0.016
ENSP00000287156	UBE2L6	152	0.017

ENSP00000269349	EIF4A3	15	0.018
ENSP00000171887	TNS1	5	0.018
ENSP00000341826	HNRNPA1	152	0.019
ENSP00000370719	ITSN1	2	0.019
ENSP00000227507	CCND1	594	0.02
ENSP00000362092	RRAGC	152	0.021
ENSP00000361366	SFTPD	152	0.022
ENSP00000310040	EIF3F	150	0.022
ENSP00000363019	UBE2D1	61	0.022
ENSP00000246551	HCST	298	0.023
ENSP00000216714	APEX1	196	0.023
ENSP00000351885	PPP2R4	152	0.023
ENSP00000227135	SPA17	2	0.023
ENSP00000324248	PENK	1	0.023
ENSP00000293308	KRT8	152	0.024
ENSP00000296695	SPINK1	152	0.024
ENSP00000377783	PROS1	152	0.024
ENSP00000356999	USF1	152	0.026
ENSP00000345530	NEDD4	152	0.029
ENSP00000327336	BGN	1	0.029
ENSP00000223215	MEST	152	0.03
ENSP00000281821	EPHA4	302	0.031

ENSP00000264110	ATF2	154	0.032
ENSP00000247970	PIN1	152	0.032
ENSP00000321706	GEMIN4	4	0.032
ENSP00000343418	SEMA4D	2	0.032
ENSP00000265563	PRKAR2A	303	0.033
ENSP00000379110	CXCL1	107	0.033
ENSP00000330341	SOCS3	27	0.033
ENSP00000306245	FOS	318	0.035
ENSP00000354280	PRSS3	152	0.037
ENSP00000228916	SCNN1A	152	0.039
ENSP00000269280	NLRP1	152	0.04
ENSP00000307863	U2AF2	152	0.04
ENSP00000357393	EFNA3	111	0.041
ENSP00000206249	ESR1	982	0.043
ENSP00000228850	AKAP3	2	0.043
ENSP00000396843	NFIC	1	0.044
ENSP00000310129	PSMD2	152	0.045
ENSP00000356248	PTPN7	150	0.045
ENSP00000339992	MYB	303	0.046
ENSP00000322542	GTF2I	152	0.046
ENSP00000341208	STAT5A	17	0.046
ENSP00000216455	PSMA3	9	0.046

ENSP00000397323	EHMT2	152	0.047
ENSP00000296504	SAP30	151	0.048
ENSP00000305480	FEN1	195	0.049
ENSP00000361186	TP53RK	152	0.049
ENSP00000363826	FZD8	152	0.049
ENSP00000338983	MUC1	147	0.05
ENSP00000352272	MYOZ1	152	0.051
ENSP00000243611	C4BPB	1	0.051
ENSP00000356771	F5	152	0.052
ENSP00000379204	BMP7	134	0.054
ENSP00000360525	MAGOH	137	0.055
ENSP00000321999	PTH1R	14	0.057
ENSP00000252487	TOMM40	2	0.057
ENSP00000268712	NCOR1	217	0.058
ENSP00000308450	CDC20	345	0.059
ENSP00000255465	CCNA1	194	0.059
ENSP00000258418	CAB39	152	0.06
ENSP00000259633	CD72	2	0.06
ENSP00000217961	STS	1	0.06
ENSP00000312624	TCAP	152	0.062
ENSP00000354125	EIF3B	151	0.062
ENSP00000313829	KHDRBS1	161	0.063

ENSP00000309871	RACGAP1	66	0.063
ENSP00000249923	COPB1	152	0.064
ENSP00000359818	MMS19	152	0.064
ENSP00000315615	AKAP5	195	0.065
ENSP00000329419	COPB2	152	0.065
ENSP00000372991	LTA	1	0.065
ENSP00000383199	NEDD4L	152	0.066
ENSP00000335153	HSP90AA1	411	0.068
ENSP00000304169	PITX2	302	0.068
ENSP00000381793	GRB10	152	0.068
ENSP00000369757	RPS6	117	0.068
ENSP00000370503	CCM2	150	0.069
ENSP00000308176	BTK	152	0.07
ENSP00000344668	KRIT1	132	0.07
ENSP00000306124	PRKCE	152	0.072
ENSP00000357177	ARHGEF11	150	0.074
ENSP00000319635	CXCR2	107	0.074
ENSP00000296509	MAD2L1	152	0.075
ENSP00000360141	GNAS	152	0.075
ENSP00000386165	CEBPD	89	0.076
ENSP00000189444	NFKB2	15	0.078
ENSP00000377446	SUCLG1	302	0.08

ENSP00000368438	PCNA	454	0.083
ENSP00000357255	BGLAP	2	0.083
ENSP00000333001	RBM8A	137	0.085
ENSP00000265056	MCM2	152	0.087
ENSP00000343745	DICER1	152	0.088
ENSP00000340766	SMG7	1	0.089
ENSP00000352516	DNMT1	169	0.093
ENSP00000261349	LRP6	302	0.094
ENSP00000262887	XRCC1	152	0.094
ENSP00000372023	CHEK2	276	0.095
ENSP00000223029	AIMP2	152	0.095
ENSP00000370557	MIS12	94	0.095
ENSP00000297439	DEFB1	149	0.096
ENSP00000304350	PRPF8	152	0.097
ENSP00000301019	CDT1	6	0.1
ENSP00000301843	CTTN	152	0.101
ENSP00000306866	GABARAP	150	0.102
ENSP00000298139	WRN	147	0.102
ENSP00000220592	AGO2	152	0.103
ENSP00000257904	CDK4	101	0.104
ENSP00000281453	MLF1IP	3	0.104
ENSP00000328777	EFNA5	18	0.105

ENSP00000387699	CREB1	540	0.106
ENSP00000305422	CEBPB	9	0.107
ENSP00000364133	TGFBR1	204	0.109
ENSP00000410076	CASP1	152	0.111
ENSP00000348307	SIRPA	63	0.116
ENSP00000276682	EIF3H	2	0.121
ENSP00000244741	CDKN1A	267	0.123
ENSP00000287727	ZFYVE9	166	0.123
ENSP00000357697	S100A2	74	0.123
ENSP00000276414	GNRH1	151	0.124
ENSP00000294339	TAL1	150	0.124
ENSP00000228872	CDKN1B	308	0.125
ENSP00000276072	TAF1	152	0.125
ENSP00000366013	GNB2L1	152	0.125
ENSP00000229595	ASF1A	152	0.127
ENSP00000349547	RASSF1	152	0.128
ENSP00000309831	SNUPN	147	0.132
ENSP00000346440	TCF4	33	0.132
ENSP00000267843	FGF7	15	0.133
ENSP00000216160	TAB1	39	0.135
ENSP00000329357	SP1	573	0.136
ENSP00000273853	CENPC1	124	0.136

ENSP00000339393	CCR6	149	0.137
ENSP00000365858	GATA1	2	0.138
ENSP00000403721	CYP21A2	152	0.14
ENSP00000355153	CDKN2A	152	0.143
ENSP00000264498	FGF2	22	0.144
ENSP00000228837	FGF6	35	0.145
ENSP00000262187	RHEB	6	0.145
ENSP00000282091	PTH	151	0.147
ENSP00000314151	KLK3	152	0.148
ENSP00000354927	MAP3K3	18	0.148
ENSP00000299402	APBB1	3	0.148
ENSP00000351671	CCL20	149	0.149
ENSP00000380921	SH3KBP1	189	0.151
ENSP00000366746	STAM	4	0.151
ENSP00000265421	POLB	151	0.152
ENSP00000354558	MTOR	418	0.153
ENSP00000336868	CENPA	149	0.154
ENSP00000258743	IL6	214	0.155
ENSP00000331358	GAST	152	0.156
ENSP00000363089	TLR4	28	0.158
ENSP00000315702	MOB4	22	0.16
ENSP00000363071	DES	11	0.165

ENSP00000285398	ERCC3	152	0.166
ENSP00000251849	RAF1	320	0.171
ENSP00000254122	FSHB	1	0.171
ENSP00000326804	CUL1	74	0.172
ENSP00000305651	CXCL10	1	0.176
ENSP00000280357	IL18	158	0.178
ENSP00000283147	BMP6	2	0.178
ENSP00000233156	TFPI	1	0.18
ENSP00000263621	ELANE	2	0.183
ENSP00000348786	RAP1A	132	0.184
ENSP00000252034	ELN	152	0.186
ENSP00000347858	XIAP	29	0.194
ENSP00000281950	GEMIN6	7	0.194
ENSP00000375777	STRN4	21	0.195
ENSP00000415941	C4B	152	0.196
ENSP00000262033	PTGES3	2	0.198
ENSP00000263253	EP300	1554	0.199
ENSP00000052754	DCN	64	0.2
ENSP00000267169	DIABLO	4	0.203
ENSP00000306512	IL8	119	0.207
ENSP00000223129	RPA3	47	0.209
ENSP00000369050	CYP1A1	151	0.212

ENSP00000239223	DUSP1	2	0.215
ENSP00000309103	BAD	270	0.216
ENSP00000358716	DDX20	151	0.217
ENSP00000344456	CTNNB1	1782	0.219
ENSP00000356016	CR1	151	0.221
ENSP00000262965	TCF3	26	0.221
ENSP00000293288	BAX	3	0.227
ENSP00000364709	F10	1	0.227
ENSP00000348577	RANGAP1	254	0.229
ENSP00000363822	AR	301	0.231
ENSP00000346437	ATG7	150	0.232
ENSP00000254719	RPA1	47	0.232
ENSP00000224337	BLNK	151	0.242
ENSP00000227163	SPI1	2	0.242
ENSP00000391592	PTPN6	155	0.246
ENSP00000361066	NCOA3	90	0.246
ENSP00000358918	SUFU	1	0.246
ENSP00000242057	AHR	76	0.247
ENSP00000379213	PTHLH	4	0.253
ENSP00000259808	RIPK1	143	0.256
ENSP00000334122	FGF3	50	0.259
ENSP00000361405	MMP9	85	0.261

ENSP00000332049	CD86	17	0.261
ENSP00000352514	RUNX2	83	0.262
ENSP00000379330	NFATC2	32	0.266
ENSP00000256443	CDK7	130	0.275
ENSP00000269321	ARHGDIA	309	0.278
ENSP00000351486	NTRK1	98	0.278
ENSP00000339527	FOXO3	157	0.279
ENSP00000331746	CALCA	130	0.279
ENSP00000288986	NCK1	92	0.279
ENSP00000264246	CD80	10	0.279
ENSP00000265734	CDK6	4	0.279
ENSP00000379625	MYD88	29	0.282
ENSP00000263025	MAPK3	13	0.285
ENSP00000276201	UPF3B	16	0.286
ENSP00000356832	SGK1	152	0.288
ENSP00000359424	CHUK	77	0.288
ENSP00000363970	MAP1LC3A	150	0.291
ENSP00000302486	MAP2K1	49	0.292
ENSP00000268035	IGF1R	111	0.294
ENSP00000345571	E2F1	160	0.296
ENSP00000332643	NDN	8	0.297
ENSP00000085219	CD22	1	0.297

ENSP00000285021	XPC	2	0.298
ENSP00000267996	TPM1	152	0.301
ENSP00000357879	PSMD4	333	0.305
ENSP00000374455	SQSTM1	150	0.305
ENSP00000262320	AXIN1	146	0.306
ENSP00000274335	PIK3R1	64	0.307
ENSP00000298171	TSHR	1	0.307
ENSP00000295731	IHH	4	0.308
ENSP00000227758	BIRC2	4	0.31
ENSP00000350720	SMARCA4	152	0.311
ENSP00000352561	CALCR	130	0.313
ENSP00000418447	PPP2CA	171	0.315
ENSP00000302530	BUB1	44	0.315
ENSP00000386259	NEB	11	0.32
ENSP00000324890	CD28	27	0.328
ENSP00000234071	PROC	1	0.329
ENSP00000263753	SGOL1	169	0.333
ENSP00000176195	SCT	130	0.336
ENSP00000296029	PF4	1	0.337
ENSP00000340944	PTPN11	223	0.34
ENSP00000355537	ACTN2	143	0.34
ENSP00000363055	ZWINT	30	0.34

ENSP00000231509	NR3C1	54	0.341
ENSP00000172229	NGFR	8	0.342
ENSP00000358595	CGA	1	0.342
ENSP00000400175	RHOA	302	0.345
ENSP00000310596	LSM1	15	0.346
ENSP00000222139	EPOR	9	0.346
ENSP00000354394	STAT1	244	0.347
ENSP00000210313	PSMD5	70	0.348
ENSP00000254227	NR0B2	152	0.349
ENSP00000367830	PRKCZ	5	0.359
ENSP00000262803	UPF1	1	0.359
ENSP00000257408	KLB	2	0.364
ENSP00000304895	IRS1	349	0.367
ENSP00000237837	FGF23	3	0.368
ENSP00000298316	ARF6	152	0.369
ENSP00000284981	APP	453	0.371
ENSP00000380280	FGFR1	222	0.374
ENSP00000358997	IRAK1	51	0.374
ENSP00000266079	PRPF6	2	0.377
ENSP00000245907	C3	151	0.378
ENSP00000339007	GRB2	1365	0.38
ENSP00000362795	CXCR3	1	0.381

ENSP00000356713	IFNGR1	21	0.387
ENSP00000263341	IL1B	80	0.388
ENSP00000209728	CDC6	105	0.39
ENSP00000311032	CASP3	7	0.401
ENSP00000299293	FRS2	137	0.404
ENSP00000350708	RAD23B	2	0.404
ENSP00000361359	CD40	11	0.405
ENSP00000294172	NXF1	136	0.409
ENSP00000309845	HRAS	212	0.414
ENSP00000352400	NUP214	136	0.422
ENSP00000256592	TSHB	1	0.422
ENSP00000356024	CR2	151	0.425
ENSP00000274255	SKP2	42	0.427
ENSP00000216797	NFKBIA	150	0.432
ENSP00000354621	SMURF1	7	0.433
ENSP00000332353	PTCH1	2	0.434
ENSP00000356438	PTGS2	152	0.44
ENSP00000384675	SOS1	119	0.441
ENSP00000326366	PSEN1	447	0.444
ENSP00000262367	CREBBP	283	0.444
ENSP00000353483	MAPK8	390	0.446
ENSP00000256897	CCNH	20	0.447

ENSP00000329380	GP1BA	150	0.449
ENSP00000300161	YWHAB	4	0.451
ENSP00000351407	ARNT	74	0.457
ENSP00000177694	TBX21	10	0.457
ENSP00000287598	BUB1B	80	0.458
ENSP00000215832	MAPK1	333	0.463
ENSP00000321656	CDC25C	383	0.465
ENSP00000396127	RAN	262	0.466
ENSP00000256442	CCNB1	77	0.469
ENSP00000268182	IQGAP1	216	0.47
ENSP00000348708	UPF2	15	0.47
ENSP00000270202	AKT1	1179	0.472
ENSP00000266970	CDK2	166	0.479
ENSP00000320940	NCOA1	35	0.479
ENSP00000264554	SHC2	5	0.481
ENSP00000340330	KAT5	62	0.483
ENSP00000277120	NTRK2	46	0.483
ENSP00000338018	HIF1A	480	0.491
ENSP00000324806	GSK3B	505	0.492
ENSP00000221972	CD79A	1	0.492
ENSP00000230354	TBP	424	0.495
ENSP00000309503	YWHAZ	171	0.495

ENSP00000387662	GCG	289	0.496
ENSP00000310491	ARHGAP1	24	0.5
ENSP00000361275	PLK3	147	0.507
ENSP00000371138	FKBP1A	75	0.51
ENSP00000293272	CCL5	41	0.511
ENSP00000267859	BNIP2	91	0.521
ENSP00000378332	TGFB1I1	3	0.523
ENSP00000354586	GLI2	1	0.523
ENSP00000262158	SMAD7	14	0.525
ENSP00000313419	CD19	151	0.526
ENSP00000308541	F2	150	0.526
ENSP00000211998	VCL	148	0.529
ENSP00000365016	IRS2	7	0.531
ENSP00000301838	FADD	9	0.537
ENSP00000353059	APAF1	124	0.539
ENSP00000324897	UBE2I	230	0.544
ENSP00000245451	BMP4	9	0.545
ENSP00000342793	PLD1	152	0.551
ENSP00000229135	IFNG	116	0.556
ENSP00000321797	FGF8	26	0.561
ENSP00000278916	CHEK1	155	0.564
ENSP00000302269	VAV1	174	0.567

ENSP00000269485	TNFRSF11A	1	0.567
ENSP00000302160	LSM3	2	0.57
ENSP00000368104	BMP2	7	0.572
ENSP00000340858	B2M	12	0.573
ENSP00000358622	IKBKG	27	0.578
ENSP00000302150	PRL	133	0.58
ENSP00000384273	RELA	285	0.584
ENSP00000371432	PRLR	133	0.586
ENSP00000330393	LEPR	12	0.613
ENSP00000360683	PTPN1	57	0.614
ENSP00000318297	RUVBL1	3	0.616
ENSP00000329623	BCL2	514	0.623
ENSP00000305769	SMAD1	149	0.624
ENSP00000267163	RB1	159	0.625
ENSP00000356425	UCHL5	86	0.625
ENSP00000302967	HDAC3	4	0.628
ENSP00000262238	YY1	4	0.639
ENSP00000219476	TSC2	41	0.655
ENSP00000348986	INS-IGF2	225	0.656
ENSP00000274376	RASA1	10	0.658
ENSP00000241014	MAPK8IP1	1	0.663
ENSP00000309597	MAP3K11	1	0.665

ENSP00000250003	MYOD1	26	0.669
ENSP00000371067	JAK2	236	0.672
ENSP00000364898	SYK	20	0.676
ENSP00000297261	SHH	2	0.682
ENSP00000223023	WASL	106	0.684
ENSP00000278568	PAK1	34	0.684
ENSP00000279593	GRIN2B	144	0.685
ENSP00000304903	CD2BP2	299	0.688
ENSP00000212015	SIRT1	81	0.69
ENSP00000264972	ZAP70	6	0.69
ENSP00000348461	RAC1	612	0.692
ENSP00000264657	STAT3	950	0.694
ENSP00000292303	CCR5	41	0.698
ENSP00000263923	KDR	9	0.702
ENSP00000314458	CDC42	349	0.703
ENSP00000312652	LEP	286	0.709
ENSP00000341551	SMAD4	158	0.71
ENSP00000344352	ATF3	79	0.712
ENSP00000376076	SUMO1	32	0.712
ENSP00000341189	PTK2	185	0.717
ENSP00000338799	IL6ST	17	0.719
ENSP00000284384	PRKCA	1	0.723

ENSP00000358490	CD2	299	0.734
ENSP00000252622	LSM7	13	0.735
ENSP00000401303	SHC1	169	0.745
ENSP00000244007	PLCG1	90	0.749
ENSP00000360266	JUN	146	0.775
ENSP00000249636	PIAS1	12	0.776
ENSP00000221930	TGFB1	67	0.782
ENSP00000215829	SNRPD3	19	0.787
ENSP00000350283	BRCA1	186	0.789
ENSP00000275493	EGFR	1030	0.79
ENSP00000312995	CLSPN	33	0.81
ENSP00000364893	ARHGEF7	48	0.818
ENSP00000162749	TNFRSF1A	5	0.823
ENSP00000222254	PIK3R2	2	0.823
ENSP00000361125	VEGFA	9	0.826
ENSP00000359206	BTRC	83	0.827
ENSP00000264033	CBL	299	0.832
ENSP00000355865	PARK2	152	0.845
ENSP00000258682	CAMK2B	81	0.845
ENSP00000399968	NCOA2	21	0.847
ENSP00000365891	WAS	10	0.847
ENSP00000011653	CD4	55	0.848

ENSP00000343204	JAK1	7	0.85
ENSP00000283635	CD8A	15	0.876
ENSP00000361626	YBX1	151	0.888
ENSP00000283195	RANBP2	8	0.894
ENSP00000337825	LCK	324	0.897
ENSP00000221494	SF3A2	315	0.898
ENSP00000339151	IKBKB	5	0.908
ENSP00000363676	RPL11	117	0.913
ENSP00000340820	MAPT	1	0.922
ENSP00000226574	NFKB1	12	0.926
ENSP00000046794	LCP2	184	0.929
ENSP00000344818	UBC	1204	0.931
ENSP00000226730	IL2	136	0.942
ENSP00000346300	CRKL	4	0.948
ENSP00000265171	EGF	2	0.948
ENSP00000361021	PTEN	76	0.951
ENSP00000162330	BCAR1	19	0.953
ENSP00000400591	SNRPE	1	0.956
ENSP00000378338	GIT1	3	0.967
ENSP00000338345	SNCA	3	0.969
ENSP00000262613	SLC9A3R1	14	0.971
ENSP00000299421	ILK	9	0.973

ENSP00000357656	FYN	64	0.979
ENSP00000228307	PXN	44	0.982
ENSP00000417281	MDM2	375	0.985
ENSP00000356346	PTPRC	15	0.993
ENSP00000300574	CRK	6	0.993
ENSP00000380227	ITGA4	2	0.995
ENSP00000346839	FN1	2	0.997
